# Vaccine hesitancy prospectively predicts nocebo side-effects following COVID-19 vaccination

**DOI:** 10.1038/s41598-022-21434-7

**Published:** 2022-12-05

**Authors:** Yaakov S. G. Hoffman, Yafit Levin, Yuval Palgi, Robin Goodwin, Menachem Ben-Ezra, Lee Greenblatt-Kimron

**Affiliations:** 1 The Interdisciplinary Department of Social Sciences, Bar-Ilan University, Ramat-Gan, Israel; 2 School of Social Work and School of Education, Ariel University, Ariel, Israel; 3 Department of Gerontology, University of Haifa, Haifa, Israel; 4 Department of Psychology, Warwick University, Coventry, UK; 5 School of Social Work, Ariel University, Ariel, Israel

**Keywords:** Psychology, Public health

## Abstract

The directionality between vaccine hesitancy and COVID-19 vaccine side-effects has not been hitherto examined. We hypothesized a nocebo effect, whereby vaccine hesitancy towards the second Pfizer vaccination dose predicts subsequent side-effects for a booster dose, beyond other effects. We expected these nocebo effects to be driven by (mis)information in males and prior experience in females. A representative sample of older adults (*n* = 756, mean age = 68.9 ± 3.43) were questioned in a typical cross-lagged design (wave 1 following a second Pfizer dose, wave 2 after their booster). As hypothesized, earlier vaccine hesitancy predicted subsequent booster side-effects for females (*β* = 0.10 *p* = 0.025, *f *^2^ = 0.02) and males (*β* = 0.34, *p* < 0.001, *f *^2^ = 0.16); effects were stronger in males (*χ*^2^*Δ* (1) = 4.34, *p* = 0.03). The (W1-to-W2) side-effect autoregression was stronger in females (*β* = .34, *p* < 0.001; males *β* = 0.18, *p* < 0.001), χ^2^Δ (1) = 26.86, *p* < 0.001. Results show that a quantifiable and meaningful portion of COVID-19 vaccine side-effects is predicted by vaccine hesitancy, demonstrating that side-effects comprise a *psychosomatic* nocebo component in vaccinated individuals. The data reveal distinct risk levels for future side-effects, suggesting the need to tailor public health messaging.

## Introduction

COVID-19 vaccine development should have been the “light at the end of the tunnel”^1^. However, this process has been hampered at critical time points by vaccine hesitancy, defined in 2014 by the World Health Organization (WHO)^2^ as “the reluctance or refusal to vaccinate despite the availability of vaccines” (p. 3). Even prior to Covid-19, the WHO considered vaccine hesitancy to be one of the 10 global threats to public health^3^. Vaccine hesitancy has become even more critical in the COVID-19 context, as vaccination prevents both the spreading of SARS-CoV-2 and decreases morbidity and mortality^4^. It is vital to distinguish vaccine hesitancy from a negative antivaxx position, even though both may lead to a declaration of vaccine refusal^5^. While vaccine hesitancy is defined^5^ as an emotional or cognitive “response to assessing the risks and benefits of vaccination” and *often results in actual vaccination* (p. 2435), antivaccination typically reflects a *refusal* to vaccinate, motivated by ideology, politics or religion^6^. Both vaccine hesitancy and refusal are impacted by considerations of safety and side-effects^7,8^. Concern over vaccine side-effects has been linked with side-effect misinformation, disseminated by social media, or even by the media (over)-profiling *accurate* but *rare* side-effects^7,9^. Experimental surveys show that vaccine hesitancy increases when participants are exposed to misinformation describing more prevalent severe side-effects^10–12^. These studies led to the notion that the prevalence of side-effects, and especially their severity, drive vaccine hesitancy^11,12^. Following this idea, messaging regarding vaccine safety is dominant in public health campaigns^9,13^.

However, several points regarding this side-effect—vaccine hesitancy link, merit attention. First, as vaccine hesitancy questionnaires include items pertaining to side-effects^14^, it is essential to show that vaccine hesitancy’s association with side-effects holds even without these items. Second, the observed relationship between side-effects and vaccine hesitancy has focused on *intent* (to vaccinate) and *concern* with *information* regarding side-effects^11,12^. As intent/concern do not typically match *actual* behavior, e.g.,^15^ one cannot necessarily discern from such studies if side-effects experienced in vaccinated persons also predict subsequent vaccine hesitancy. Third, even if such studies had been based on actual behavior, the obtained data would not preclude the opposite direction of vaccine hesitancy predicting future side-effects. This latter direction essentially reflects a nocebo effect^16^, whereby adverse vaccine side-effects are predicted by one’s prior negative attitude towards the vaccine. The primary goal of the current research was to discern the directionality of the vaccine hesitancy—side-effect link. Discerning directionality requires the application of a cross-lagged design whereby both vaccine hesitancy and side-effects are examined at two different time points (W1 and W2). In the following paragraph we outline the underlying rationale for this latter direction (of vaccine hesitancy predicting side-effects) which constitutes a nocebo effect.

Nocebo effects can be shown in clinical trials, such as the COVID-19 Pfizer trials, whereby participants are typically randomly allocated into one of two groups. In one group participants received the COVID-19 Pfizer vaccination (treatment group); in the other group participants did not receive treatment but rather a placebo (placebo condition). The clinical COVID-19 Pfizer vaccine trials show that even participants in the untreated group exhibited vaccination side-effects^16,17^. Nocebo effects can also be demonstrated in a variety of laboratory studies and experimental designs^18^. Several factors impact nocebo effects. For example, negative affect, e.g., persons suffering from general anxiety may display more nocebo side-effects^19^. Another central factor that affects nocebo effects is one’s past learning experience (e.g., conditioning), even if this is limited to a single learning event^20^. Perhaps the most central factor that impacts one’s nocebo effect is prior negative expectations^18^, which are defined as specific negative cognitions e.g., when participants expect to feel tired, pain or nausea after vaccination, they are more likely to suffer from these side-effects after vaccination. Expectations can also stem from exposure to (mis)information^21^ or prior experience^20^. Notably, even general negative expectations or attitudes, such as the personality trait of pessimism, also link with greater nocebo effects^22^. In this context, like negative expectations and pessimism, vaccine hesitancy, a negative cognitive and emotional response to the vaccine^5^ stemming from (mis)information^7,8^, should also predict subsequent W2 side-effects. Vaccine hesitancy also includes items that explicitly address side-effect expectations (e.g., I am concerned about immediate negative vaccine side-effects “). Accordingly, as stated above, it is important to show that even the non-expectation vaccine hesitancy items predict subsequent W2 side-effects. As vaccine hesitancy was hitherto examined in unvaccinated persons, showing that it predicts vaccine side-effects in vaccinated persons constitutes a novel perspective.

This nocebo effect can be further strengthened by the emergence of typical sex differences which were found in experimental studies, whereby males display greater nocebo effects than females following manipulations of (mis)information impacting expectations. In contrast, females display greater nocebo effects following actual experience^23,24^. It was suggested that these effects emerge more readily in laboratory studies as there is a distinct delineation of prior experience and negative expectations^24^. Such delineated separation is part and parcel of the current cross-lagged design, where prior experience (e.g., W1 side-effects) and one’s negative attitude of vaccine hesitancy (impacted by (mis)information) are distinctly separated. Accordingly, these sex differences should also be evident in the current cross-lagged design.

In addition to assessing the primary goal of directionality, another important goal was to estimate the magnitude of this effect in vaccinated persons. Even in COVID-19 clinical trials where a nocebo effect was shown^16,17^, it is not trivial to estimate the level of nocebo side-effects in vaccinated persons, from side-effect levels observed in the placebo condition. Such an estimation would require the statistical assumption that nocebo effects are additive (i.e., constant in both the vaccinated and placebo groups), an assumption that has been challenged^25,26^. The current cross-lagged design, however, should enable assessing the magnitude of side-effects predicted by the psychological variable of vaccine hesitancy in vaccinated persons. To the best of our knowledge, only a single study has addressed how psychological factors predict COVID-19 vaccine side effects in vaccinated persons^27^. In that study, pre-vaccination psychological factors (vaccine side-effect expectations, COVID-19 worries, and depressive symptoms) predicted seven post-vaccination side-effects—(1) pain at injection site, (2) fever, (3) chills, (4) headache, (5) joint pain, (6) nausea, (7) fatigue, which include the three side-effects reported in Pfizer clinical trials^28^, i.e., headaches, fatigue, and pain at injection site. While that study^27^ is a significant step in the current direction, the following points render it somewhat less relevant to the issue at hand. First, as a cross-lagged design was not employed in that study, directionality could not be discerned. Thus, the obtained result of psychological factors predicting side-effects was not shown to exist beyond other potential effects, such as the opposite direction of side-effects predicting subsequent psychological factors. Moreover, the number of side-effects was quite limited^27^. Finally, vaccine hesitancy was also not assessed.

Following the above, the current study employed a suitable cross-lagged design to examine the directionality of the vaccine hesitancy—vaccine side-effect link. Vaccine hesitancy and side-effects were assessed (at W1 and W2) in a representative sample of older adults who face the greatest risk from COVID-19 complications and thus have the highest vaccination rate^29^. This in turn enables assessment of vaccine hesitancy levels amongst *vaccinated persons*. Another advantage of focusing on this age group is that side-effect levels are typically lower in older adults^30^, rendering the detection of such effects more challenging.

Aligned with the nocebo conceptualization, it is hypothesized that W1 vaccine hesitancy would predict W2 booster side-effects. Sex differences are also predicted based on earlier findings whereby nocebo effects are driven more by mis/information for males and more strongly by learning (i.e., previous experience) for females^23,24^. Accordingly, as vaccine hesitancy is based on (mis)information^6^, the hypothesized linking of prior vaccine hesitancy with subsequent vaccine side-effects should be more robust in males than in females. Moreover, in line with nocebo effects being driven by actual experience in females, the experiencing of side-effects at W1 should predict W2 side-effects more robustly in females than in males. These expected results should be independent of the higher vaccine hesitancy levels^31^, and larger side-effect levels^32^ observed in females. Supplementary analyses include models that examine this hypothesized direction across different side-effects and different vaccine hesitancy items (those addressing side-effect expectations and those that do not). A final supplementary model includes (W1 and W2) general anxiety and a single W2 item addressing a general expectation. The goal of these supplementary analyses was to examine if the hypothesized nocebo effects hold beyond these items and variables.

## Results

### Descriptive statistics

Descriptive statistics of demographic variables appear in Supplementary Table S1. Severity of each side-effect is depicted separately for each wave in the Supplementary Table S2. As in other studies^1,^ severe side-effects were rare. Table 1 displays descriptive statistics regarding the main study measures and their W1-W2 intercorrelations, along with presenting the correlations between these main variables and demographic variables. Vaccine hesitancy was positively associated with side-effect severity both within wave and across wave.

**Table 1:** Means, Standard Deviations, and Intercorrelations Between Main Study Measures.

Variable	1	2	3	4	5	6	7
1. Age	–						
2. Education	.01	–					
3. Sex	− 0.02	− 0.02	–				
4. W1 Hesitancy	0.19***	0.09*	0.12**	–			
5. W2 Hesitancy	− 0.08*	0.11	0.14**	0.54***	–		
6. W1 Side-effects	− 0.09*	0.03	0.08*	0.22***	0.12***	–	
7. W2 Side-effects	− 0.08*	− 0.06	0.22***	0.25***	0.38***	0.31***	–
M	69.80	5.18	−	1.93	1.77	1.25	1.26
SD	3.47	0.94	−	0.67	0.61	0.33	0.35

*N* = 756, ∗ *p* < 0.05, ∗  ∗ *p* < 0.01, ∗  ∗  ∗ *p* < 0.001. All correlations are Pearson, except for Sex which is point bi-serial.

To assess the effects of time (W1 and W2) and sex (males vs. females) on vaccine hesitancy, a repeated measures ANOVA was conducted. The interaction was not significant (see Table 2). Yet both main effects were significant, i.e., females reported higher vaccine hesitancy, and vaccine hesitancy overall decreased across waves (see Table 2). A similar analysis on side-effects revealed a 2 wave (W1 vs. W2) X 2 sex (males vs. females) interaction. While side-effects decreased across waves for males, they increased for females (Table 2). As shown in Table 2, only the sex main effect was significant, i.e., females had higher side-effect levels than males at both waves. Yet side-effects remained stable across waves (Table 2).

**Table 2:** Time and Sex main effects and their interaction on vaccine hesitancy and on side-effects based on Repeated measure ANOVA analyses.

Variable	Males	Females	Sex main effect F (1754) =	*p*	ŋ^2^	Time main effects F (1754) =	*p*	ŋ^2^	Time*Sex Interaction effect F (1754) =	*p*	ŋ^2^
Hesitancy W1	1.83 (0.63)	1.99 (0.69)	3.23**	= 0.001	.15	47.21***	< 0.001	0.05	0.09 n.s	0.824	0.00
Hesitancy W2	1.67 (0.57)	1.84 (0.62)	3.87***	< 0.001	0.16						
Side effects W1	1.21 (0.38)	1.27 (0.28)	2.15*	0.032	0.05	0.00	0.978	0.00	11.62***	< 0.001	0.03
Side effects W2	1.16 (0.23)	1.32 (0.39)	6.04***	< 0.001	0.15						

*N* = 756, ∗ *p* < 0.05, ∗  ∗ *p* < 0.01, ∗  ∗  ∗ *p* < 0.001.

### Main model

**Figure 1:**
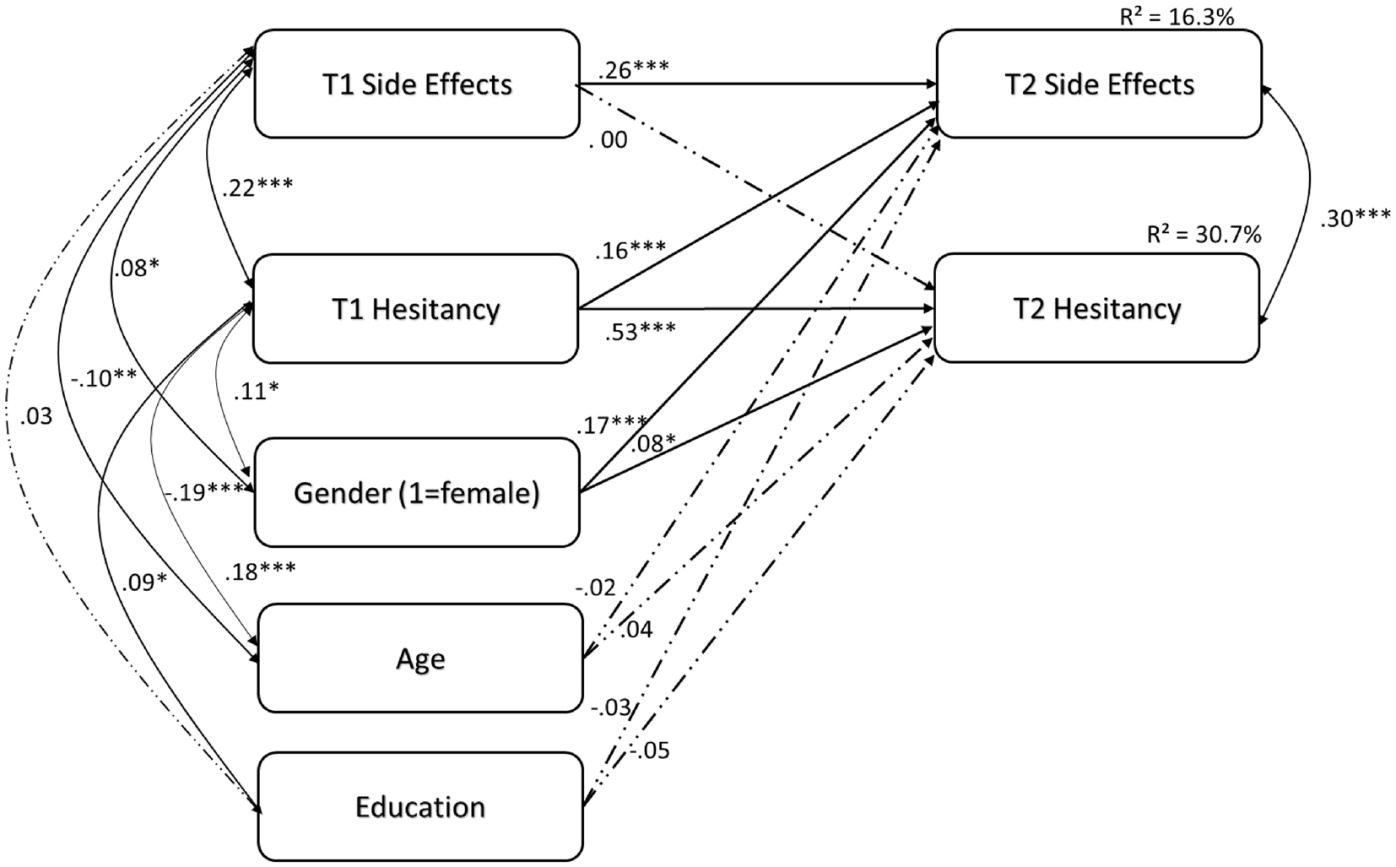
Cross-lagged associations between vaccine hesitancy and vaccine side-effects. *Notes N* = 756, ∗ *p* < 0.05, ∗  ∗ *p* < 0.01, ∗  ∗  ∗ *p* < 0.001.

Goodness of fit for the cross-lagged path model was excellent: χ^2^ (df = 2, *N* = 756) = 0.44, *p* = 0.801; RMSEA = 0.03 (90% CI = 0.000, 0.065; CFI = 1.00; SRMR = 0.010). Figure 1 shows the standardized coefficients of the model, and its different effects (stable autoregressive and cross-lagged paths). Statistically significant lagged effects emerged from W1 side-effects to W2 side-effects (*β* = 0.26, *p* < 0.001), and from W1 vaccine hesitancy to W2 vaccine hesitancy (*β* = 0.53, *p* < 0.001). As for cross effects, only the direction of higher W1 vaccine hesitancy levels predicting higher W2 side-effect severity, was significant (*β* = 0.16, *p* < 0.001, *f *^2^ = 0.04, a meaningful effect size in cross-lagged designs^33^). There was no effect for W1 side-effects predicting subsequent W2 vaccine hesitancy (*p* = 0.945).

Age and education were neither associated with W2 vaccine hesitancy nor side-effects (*p’s* > 0.05). However, sex was significantly associated both with W2 vaccine hesitancy (*β* = 0.08, *p* = 0.009) and side-effects (*β* = 0.17, *p* < 0.001). Females reported higher side-effect severity levels and higher vaccine hesitancy levels compared to males (Table 2).

### Sex multi-group model

**Figure 2:**
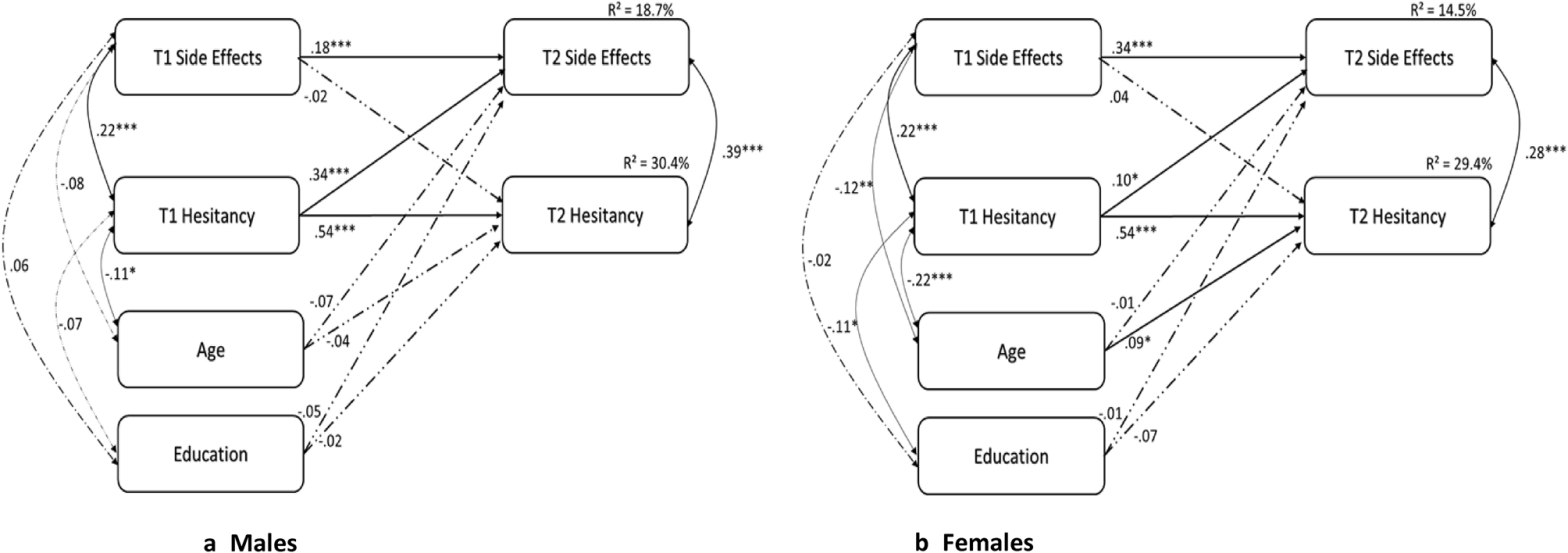
The Multi-Group Cross-lagged associations between vaccine hesitancy and vaccine side-effects for Males (**a**) and Females (**b**). *Notes N* = 756, ∗ *p* < 0.05, ∗  ∗ *p* < 0.01, ∗  ∗  ∗ *p* < 0.001.

Model parameters for a multi-group (males vs. females) cross-lagged model are presented in Table 3. Goodness of fit for this multi-group cross-lagged path model was excellent: χ^2^ (df = 2, *N* = 756) = 4.41, *p* = 0.110; RMSEA = 0.040 (90% CI = 0.000, 0.091; CFI = 0.996; SRMR = 0.010. Figure 2 shows the standardized model coefficients separately for males (Fig. 2a) and females (Fig. 2b). Statistically significant lagged effects emerged from W1-to-W2 side-effects (males: *β* = 0.18, *p* < 0.001; females: *β* = 0.34, *p* < 0.001), with this effect being more robust for females than males (χ^2^Δ (1) = 26.86, *p* < 0.001). The lagged effects observed from W1-to-W2 vaccine hesitancy was similar in males (*β* = 0.54, *p* < 0.001), and females (*β* = 0.54, *p* < 0.001).

**Table 3:** Parameters for the cross-lagged models *n* = 756.

	Males *n* = 295				Females *n* = 461			
Parameter	Standardized estimate	Lower 95%	Upper 95%	*p*	Standardized estimate	Lower 95%	Upper 95%	*p*
**Regression weights**								
W2 Side-effects ← W1 Side-effects	0.181**	0.047	0.389	0.009	0.342**	0.254	0.437	0.010
W2 Hesitancy ← W1 Hesitancy	0.540**	0.450	0.624	0.007	0.539**	0.467	0.606	0.010
W2 Hesitancy ← W1 Side-effects	− 0.022	− 0.096	0.080	0.716	0.020	− 0.054	0.091	0.584
W2 Side-effects ← W1 Hesitancy	0.336**	0.448	0.223	0.008	0.102*	0.017	0.199	0.047
**Covariates**								
W2 Side Effects ← Education	− 0.066	− 0.150	0.015	0.212	− 0.040	− 0.113	0.031	0.420
W2 Hesitancy ← Education	− 0.042	− 0.142	0.078	0.769	− 0.066	− 0.136	− 0.005	0.090
W2 Side-effects ← Age	− 0.056	− 0.113	0.032	0.239	− 0.001	− 0.063	0.053	0.942
W2 Hesitancy ← Age	− 0.061	− 0.135	0.010	0.175	0.071*	0.010	0.133	0.040

***p < 0.001 **p < 0.01 *p < 0.05.

For both sexes there was a significant cross effect for higher W1 vaccine hesitancy levels predicting subsequent W2 side-effect severity (males *β* = 0.34, *p* < 0.001, *f *^2^ = 0.16; females *β* = 0.10 *p* = 0.025, *f *^2^ = 0.02). However, as predicted, this cross effect was significantly stronger in males (*χ*^2^*Δ* (1) = 4.34, *p* = 0.03). W1 side-effects did not predict W2 vaccine hesitancy in either males or females (*p’s* > 0.05). The results (*only* hesitancy predicted subsequent side-effects) held across different follow-up supplementary models. First, they held for both typical and less typical side-effects (Supplementary Tables S3 and S4). Second, results held for vaccine hesitancy items addressing expectations and more importantly even for hesitancy items that did not address expectations (Supplementary Tables S5 and S6). (3) Results also held when general anxiety disorder (GAD-7) symptoms were included along with a (W2) single item expectation measure (Supplementary Table S7).

## Discussion

Unique contiguous waves of COVID-19 vaccination present a rare opportunity to test the directionality between vaccine hesitancy and actual vaccine side-effects. This allows for the novel empirical evaluation of the link between prior vaccine hesitancy to an earlier dose and subsequent side-effects from a later dose—instead of the previously researched link between *theoretical* side-effect information with *intent* to vaccinate. Our study had three main results. First, only prior vaccine hesitancy predicted subsequent vaccine side-effects, not vice-versa. Second, while these effects were evident for both sexes they were more robust in males (females *F*^2^ = 0.02, males, *F*^2^ = 0.16). Third, the association between W1-to-W2 side-effects was more robust in females. These latter two results are aligned with laboratory findings showing sex differences in nocebo effects^23,24^, which are driven in males more by (mis)information, and in females more by previous learning and experience. Taken together these findings indicate that in addition to previously observed nocebo effects in placebo arms of clinical trials^17^, vaccine side-effects after actual vaccination comprise an identifiable nocebo component of small-to-moderate magnitude. In cross-lagged designs, even small cross effect sizes are meaningful, as they reveal effects beyond other effects, e.g., beyond the robust W1-to-W2 autoregressive vaccine hesitancy association^33^. Had the vaccine hesitancy autoregressive link been weaker (i.e., had vaccine hesitancy been less stable across time), it would have most likely allowed the emergence of a stronger cross-effect between W1-vaccine hesitancy to W2-side-effects.

Results from previous studies have indicated that side-effect severity leads to vaccine hesitancy. This supports a generic public-health safety messaging^9,13^. From this perspective, the current results showing the opposite direction, namely that vaccination side-effects are predicted by vaccine hesitancy, highlights the following issues. First, the previously obtained direction that (concern over) side-effects predict (intent to) vaccinate^11,12^ was not significant in any of our analyses, i.e., neither for males or females (as shown Fig. 2), nor across the different side-effects, vaccine hesitancy items and anxiety (see Supplementary Table S3–S7). Instead, in line with the nocebo concept^25–27^, only the hypothesized vaccine-hesitancy → side-effects direction was supported across all analyses. These results echo the distinction between *intent* vs. *behavior*^15^, suggesting that vaccinated vs. unvaccinated individuals may constitute different groups vis-à-vis vaccine hesitancy. Secondly, vaccine hesitancy was previously considered a major threat to public health, as it rendered *those* with high hesitancy *less* likely to vaccinate. The current results however reveal a novel aspect of this threat. As vaccine hesitancy predicts increased side-effects, which in turn fuel antivaxx campaigns in their goal of deterring the wider public from vaccination^7,8^, the impact of vaccine hesitancy may extend to a wider public, beyond those with high hesitancy. Third, the psychosomatic aspect of vaccination side-effects revealed in our study has three further implications. First it suggests that a prevalent negative vaccine hesitancy attitude not only influences one’s decision to vaccinate, but also perhaps the level of side-effects one will experience after vaccination. Hitherto, most public health campaigns have focused their messaging on reducing vaccine hesitancy in unvaccinated individuals (to increase vaccination). The current results suggest that decreasing vaccine hesitancy levels is warranted in vaccinated persons as well, as it should lead to lower side-effect levels. Second, our results revealing a nocebo component of these side-effects suggest that these side-effects may be directly ameliorated by a variety of specific interventions that have been documented to reduce nocebo effects^18^; e.g., interventions that decrease one’s anxiety^19^, interventions that include different forms of conditioning to induce more positive expectations^34^ or methods that challenge negative expectations, such as pre-exposure^35^. In addition, the same information, which is typically framed negatively, can be communicated positively; such positive framing also reduces nocebo effects^36,37^. For example, instead of messages conveying that severe side-effects are rare (negative framing), it may be preferable to apply positive framing, e.g., that the vast majority of people do not experience even moderate side-effects (Table S2). Moreover, supplying more accurate information would also lower expectations and reduce nocebo effects. Third, the *psychosomatic* element of side-effects may need to be incorporated into public health messaging to reduce the nocebo element^36–39^. Essentially, the current results suggest that public health messaging may be effectively tailored to side-effect risk level, to nocebo risk level, and to vaccination status.

This latter point would require differentiated public health messaging as suggested in previous studies^9,40^. Our data importantly suggest *different risk levels for side-effects*. Low risk individuals (e.g., low vaccine hesitancy, older adults, males) can be made aware of their profile and status and be targeted with a personalized public health campaign stressing that, for *them*, the vaccine is even safer than the standard safety level for the population at large. As the cost of vaccination is low for this group, it may be less imperative to stress the benefits of vaccination. For those at higher risk for side-effects (e.g., high vaccine hesitancy, younger adults, females), a different public health messaging may be needed. Namely, in addition to acknowledging their elevated risk, *high risk persons would benefit from receiving messages stressing that a meaningful portion of their physical side-effects is psychosomatic*.

Such messaging may require caution, as although it may likely be effective in lowering nocebo effect levels^36,38^, this information in-itself may be perceived as blaming or insulting^39^, causing a defensive and less cooperative response. Thus, it is imperative that this type of messaging be accompanied by nocebo education^39^. One essential goal of nocebo education in the current context may likely be the rooting out of a common misconception that such side-effects may be conceived as “fake”. It is important for health officials to stress that nocebo effects are as physically real as any other side-effects, while at the same time conveying that they may be due to factors other than the treatment; thus, the treatment itself may not be harmful^41^. Another aspect of this issue pertains to hi-risk persons, such as females who are at higher risk for side-effects^1^ and perhaps for having their symptoms or pain taken less seriously due to gender bias^42^. Thus, an additional goal of such education would be to explain what a nocebo effect actually is (as opposed to the prior goal which focuses on what it is not). Therefore, messaging should convey that different side effects may have different causes, some may be due to the treatment itself, others may be due to issues like one’s *anxiety level about the treatment*, one’s *previous experience* or one’s *negative expectations.* Considering the above, nocebo education should promote non-threatening, soft mannered and respectful communication regarding what nocebo effects are, and what they are not. It should further address its causes and suggest potential interventions. As mentioned, if messaging that one’s side-effects likely constitute a nocebo component is guided by such nocebo education, it should facilitate reduction of side-effects due to the nocebo component. Decreasing side-effect levels would not only help on an induvial basis by alleviating the level of suffering, it may gradually change public health opinion about vaccination safety.

Finally, it should be recognized that vaccinated persons include both fully vaccinated and partially vaccinated persons. Both groups will likely require future vaccination, either because future boosters become necessary or because they did not complete their vaccination. In the USA alone, over 150 million persons received the first dose but not the booster dose (22 August, 2022, https://covid.cdc.gov/covid-data-tracker/#vaccinations_vacc-people-additional-dose-totalpop). As results suggest, vaccinated persons constitute a separate group i.e., their direct experience with vaccine side-effects may render *generic* safety messaging less relevant. To encourage further vaccination for such persons, it may be important to focus on side-effects. As also indicated by our results, it may be particularly significant to convey to males that side-effects decrease from wave-to-wave and that there is but a weak side-effect autoregression (as shown in Fig. 2a). For females however, as side-effects both increased from wave-to-wave and demonstrated stronger autorepression (W1-to-W2 side-effects, as shown in Fig. 2b), such messaging is less appropriate. Instead, it may be beneficial to acknowledge their elevated level of side-effects and the increased dependency of current side-effects on previous side-effects. Messaging could thus focus both on the fact that this elevated autoregression may likely reflect a nocebo effect^38,39^, and on nocebo education^39^. This kind of differentiated messaging is in line with research suggesting the importance of using different public health messaging about COVID-19 vaccination to different groups^40^. Pending further research, it may be helpful to develop an official interactive website where persons can enter relevant demographic data, as well as filling out a vaccine hesitancy assessment and, if relevant, report their side-effect severity to previous doses. In turn, the user can then be informed of their side-effect risk level and the estimated psychosomatic nocebo component. Such a strategy may also take the “wind out of the antivaxx propaganda sails”, thereby encouraging vaccination.

This study has several limitations. First, generalizability may be limited by the study’s focus on older adults vaccinated with the Pfizer vaccination. It is important to directly examine this issue in younger participants and with other vaccines. Second, as in many other vaccine studies, side-effects were based on self-report. Third, as vaccine hesitancy is impacted by culture^8^ and ethnic groups^40^, it is important to address this link across cultures and different ethnic groups. Fourth, although directionality was ascertained in the current study, future research would benefit from an experimental intervention to establish causality (for example, by experimentally modifying one’s vaccine hesitancy and assessing its subsequent impact on actual vaccine side-effects in a cross-lagged design). Fifth, this study focused specifically on the directionality between the critical variable of vaccine hesitancy and vaccination side effects. As our primary goal was to examine if the critical vaccine hesitancy variable (even when it does not include expectations items) predicts subsequent-side-effects, we did not focus on specific side-effect expectations and thus did not include such questionnaires^43^. To somewhat mitigate this concern, we ran additional analyses, e.g., a model analyzing the three vaccine hesitancy items which address expectation of future side effects (alongside other models see supplementary Tables S5 and S6). Future studies however could profitably address specific negative expectations. Another possible limitation is that the time duration could have yielded unwarranted variance. Namely, as boosters began on August 2022, and as W2 questioning was conducted between September 30th–October 28th, participants’ may have responded up to three months after the booster. Thus, it is possible that while some W2 participants responded to a recent booster vaccination, others may have been reporting from memory of a more distant vaccination experience. Perhaps such memory was less precise and may not sufficiently differentiate between the 3 separate COVID-19 vaccine experiences.

This study also has several strengths. First, this study is the first to introduce a cross-lagged design to assess the directionality of vaccine hesitancy and subsequent COVID-19 vaccination side-effect over a relatively long interval of six months. Second, a representative sample of older Israeli adults was assessed. Third, results indicating the nocebo effect were further supported by expected sex differences. Fourth, effect sizes were meaningful^33^ and the results were reliable in that they did not depend on specific side-effects or specific vaccine hesitancy items (see Supplementary results). As the vaccine hesitancy baseline is low in Israel^44^, the magnitude of this nocebo effect would have been likely greater in other countries. Our design enabled measuring the magnitude of psychosomatic components in COVID-19 vaccination side-effects, above and beyond other effects. This is important as, relative to placebo effects, the nocebo effect remains relatively understudied^18^.

## Method

### Participants

This study is part of an ongoing longitudinal study^32^ pertaining to vaccine side-effects in a representative sample of Israeli older adults. All data collection was conducted in accordance with the relevant guidelines and regulations. Procedures were approved by the Social Science Faculty Review Board at the final author's university. Participants indicated informed consent to procedures approved by this author’s University Institutional Review Board. At Wave 1 (January- February, 2021), 931 out of 1007 participants were vaccinated with the second Pfizer dose. At Wave 2 (September–October, 2021) 790 participants responded. Participants who contracted COVID-19 (*n* = 3) or who did not vaccinate 3 times (*n* = 31) were removed, leaving 756 participants (60.9% females, 47.4% with academic education, 74% married); for additional descriptive statistics of demographic variables, please see Supplementary Table S1.

Participants were recruited via iPanel, a probability-based panel with over 100,000 adult members who consented to be contacted about surveys^32,45^. This panel conducts studies according to the Strengthening the Reporting of Observational Studies in Epidemiology guidelines for observational studies (STROBE). The panel consists of adults aged 18–85 who have given their consent to be contacted about surveys. Panel recruitment is dynamic and constant using a range of online methods. iPanel adheres to the stringent standards of the world association for market, social and opinion researchers (ESOMAR). We used this panel to recruit participants aged 60 and above. A quota sampling approach was used with quotas meeting the Israeli national census data on variables representative of this age group, as specified by the Israeli Bureau of Statistics census data. The use of this approach ensured representation of the older adult population in Israel. After the quotas and required sample size were reached, the survey was closed. The final data set was weighted to enable the study to be considered representative of the internet-using participants aged 60 and above years living in Israel.

Although the current sample size was determined by practical considerations for obtaining a representative sample, a post-hoc power analysis revealed that the sample size (*n* = 756) had ample power to detect effects. Following established guidelines^46^, we used the RAMpath *R* package to perform a Monte Carlo power analysis based on a small effect size of *d* = 0.20, with a significance level of 0.05 for the cross-lagged effects. Running 1,000 replications per condition revealed power of 0.975 to detect significant cross-lagged effects (95% confidence intervals 0.90–1.00).

Attrition analyses revealed no group differences between participants who participated at both waves, compared to those who only participated at W1. Individual t-tests revealed that participants who participated at both waves vs. participants who only participated at W1, were similar on W1 measures of age, vaccine hesitancy, and side-effects (*p*’s = 0.099 to 0.850). Likewise, Chi square analyses revealed that these groups were similar on W1 measures of family status, sex and education (*p*’s = 0.109 to 0.945).

Participants indicated sex, education, and marital status. Side-effect severity was rated on a scale from 1-not-suffering-at-all to 5-suffering-very-severely^32^ across a list of 21 vaccine side-effects, based on information from the FDA (https://www.fda.gov/media/144414) and Israeli Ministry of Health (https://en.globes.co.il/en/article-covid-vaccineside-effects-in-israel-matchtrials-1001359338). The side-effect scale’s α was .88 and .89 at each wave respectively. Side effects included (1) Swollen arm/pain injection site, (2) Fever, (3) Chills, (4) Headaches, (5) Joint pains, (6) Nausea, (7) Feeling tired/fatigue, (8) Facial paralysis, (9) Vomiting, (10) Allergic reactions, (11) Swollen lymph nodes, (12) Rash, (13) Swollen eyes, (14) Sore throat, (15) Coughing, (16) Stomach pain, (17) Dizziness, (18) Flu-like symptoms, (19) Sleep problems, (20) Weakness, (21) Muscular pain. Vaccine hesitancy was assessed by an 8-item questionnaire^14^; α's were .83 and .84, respectively. The vaccine hesitancy items appear in the supplementary Methods section. Table S2 provides the percent of each side-effect endorsed at each severity level at both waves, as well as two dichotomous measures of vaccine side-effects (obtained by collapsing severity levels into no-side effects vs. yes-side-effects). At each wave participants were asked how many vaccines they received. As mentioned, we analyzed data only from participants who received all 3 vaccines (*n* = 756). Participants were further questioned at each wave about the extent to which they suffered from side-effects after their relevant vaccine. For example, following the booster vaccine, participants were asked about each side-effect from the third vaccine (e.g., please rate the severity of the tiredness you felt following the booster vaccine). This was stressed also for each of the five response ratings (e.g., I did not feel this side-effect of tiredness at all after the third vaccine). Supplementary analyses included a single general expectancy item administered at W2 (“I believe that the vaccine will protect me from COVID-19”, rated on a 5-point Likert scale 1-not at all to 5 -very much). In addition, we included a 7-item measure for general anxiety disorder (GAD-7) symptoms^47^, α’s for this scale were .95 and .93, respectively. There were no missing values.

### Data analytic strategy

We employed a mixed two-way repeated measures ANOVA, with a fixed factor (sex) and a repeated measure factor (time). An autoregressive cross-lagged model (ARCL) was used to explore structural relations of repeatedly measured constructs to estimate the variables’ precedence and directional influence on each other over time^48^. ARCL models were conducted in the overall sample as well as a multigroup (sex) based analysis. Goodness of fit indices, i.e., Comparative Fit Index (CFI), root mean square error of approximation (RMSEA), and standardized root mean residual (SRMR)), were computed^49^. Chi-square (χ^2^) values were cautiously interpreted given their sensitivity to sample size^50^. Cohen’s *f *^2^ effect size was calculated for significant cross-lagged effects^51^.

### Ethical approval

This study protocol was reviewed and approved by the Ariel University Institutional Review Board approval number [AU-SOC-LG-20210824].

### Consent to participate statement

Participants electronically indicated their consent to participate.

## Supplementary Information

Supplementary Information.

## Data Availability

De-identified participant data will be available in anonymized form, from the corresponding author, YL, and MB on reasonable request with an appropriate data sharing agreement, subject to review, following the publication of results.
