# Supplementary material for: Vaccine hesitancy prospectively predicts nocebo side-effects following COVID-19 vaccination
Source: Sci Rep. 2022 Dec 5;12:20018. doi: 10.1038/s41598-022-21434-7 (PMC9722845; doi:10.1038/s41598-022-21434-7)
Supplement: Supplementary file 1 — Supplementary Information. [file 41598_2022_21434_MOESM1_ESM.pdf]

Supplementary material

Vaccine hesitancy prospectively predicts nocebo side-effects following COVID-19 vaccination

Hoffman, Levin, Palgi, Goodwin, Ben-Ezra & Greenblatt-Kimron

Table of Contents

Methods **2**

Table S1 (Demographics) **2**

Table S2 (Side-effect severity) **3**

Additional Analysis addressing vaccine side-effects **4**

Table S3 (Model results for 7 side-effects [based on Geers et al., 2021] vs. remaining side-effects) **5**

Table S4 (Model results for 3 side-effects [based on COVID-19 trials] vs. remaining side-effects) **6**

Additional analyses addressing vaccine hesitancy items **7**

Table S5 (Model results without hesitancy items related to side-effects) **7**

Table S6 (Model results for vaccine hesitancy items only pertaining to side-effects) **8**

Table S7 (Model results with single W2 expectancy item and general anxiety) **9**

References **10**

Methods

Table S1. Descriptive statistics of demographic variables

Variable	Values	
**Age M(SD)**		69.80 (3.47)
**Gender**	Men	295 (39.0%)
	Women	461 (61.0%)
**Marital status**	Not in relationship	22 (2.9%)
	Married/in prolonged relationships	564 (74.6%)
	Separated/divorced	105 (13.9%)
	Widowed	65 (8.6%)
**Education**		
	Elementary school	4 (0.5%)
	Partial high school	43 (5.7%)
	Graduated high school	126 (16.7%)
	High education, non academic	224 (29.6%)
	Academic education	359 (47.5%)

Vaccine hesitancy

The eight vaccine-hesitancy items were adapted from the Giambi et al. [1] vaccine hesitancy questionnaire and were used in prior COVID-19 studies [2]. This questionnaire included the following 8 items. 1) I am concerned about immediate negative vaccine side-effects; 2) I am concerned about long-term damage due to the vaccine; 3) The Covid-19 vaccine may be more dangerous than COVID-19 itself; 4) The need to vaccinate is driven by economic interests of the pharmaceutical companies; 5) The health authorities are exposing us to positive information about the vaccine but not its dangers; 6) Vaccination is not necessary, provided you live a healthy life and follow the health regulations concerning corona; 7) Vaccination is not necessary, since COVID-19 is not as dangerous as people say it is; 8) The vaccine will impair our immune system or cause an overload.

Side-effect severity

The percentages of endorsing each severity rating for each of the 21 side-effects, at each wave are depicted in Table S2. In order to render these results comparable to studies that used a dichotomized measure of severity, we also present the frequencies of two dichotomized scores. The first dichotomized score (first column in table) was computed by collapsing percentages across severity ratings in the following manner: persons endorsing the severity level "none" and "a-little" (levels 1 and 2) were grouped together as **not** having suffered, vs. all other levels (from levels 3-moderate to 5-very severe). The second dichotomized score was more liberal at estimating side-effects, as it divided those who did not experience side-effects at all (rating of 1-none) vs. all other severity levels (ratings 2-a little – 5-very severely). See first two columns in Table S2.

Table S2. Percentage of participants (n= 756) endorsing each severity rating for each Pfizer vaccine side-effect, at W1 (left of diagonal line) and W2 (right of diagonal line). The first two columns depict dichotomized scores collapsed across severity levels.

	% side-effects (by dichotomizing scores)		% of side-effects at each severity level				
	% Persons who experienced this side-effect (moderate to very severe)	% Persons who experienced this side-effect (a little to very severe)	Not at all	A Little	Moderate	Severe	Very Severe
**Facial paralysis**	0.5/0.4	1.2/1.3	99.04 / 98.7	0.53 / 0.90	0.21 / 0.30	0.00 / 0.00	0.21 / 0.1
**Vomiting**	0.5/0.3	1.7/1.1	98.19 / 98.9	1.28 / 0.80	0.32 / 0.30	0.00 / 0.00	0.21 / 0.00
**Allergic reaction**	0.9/0.5	2.1/2.0	97.76 / 98.00	1.38 / 1.50	0.43 / 0.40	0.21 / 0.00	0.21 / 0.1
**Swollen lymph nodes**	0.9/1.1	2.9/3.6	97.02 / 96.40	2.02 / 2.50	0.43 / 0.90	0.32 / 0.00	0.21 / 0.1
**Rash**	0.9/0.5	2.9/1.7	97.34 / 98.30	1.81 / 1.20	0.43 / 0.40	0.21 / 0.10	0.21 / 0.00
**Swollen eyes**	1.6/0.9	5.3/4.4	94.78 / 95.60	3.73 / 3.40	0.96 / 0.90	0.11 / 0.00	0.43 / 0.00
**Fever**	1.9/5.6	6.9/11.2	92.86 / 88.8	5.01 / 5.70	1.28 / 3.00	0.43 / 1.10	0.43 / 1.5
**Tickling throat**	1.6/1.6	9.8/6.3	90.42 / 93.70	8.20 / 4.80	0.75 / 1.50	0.21 / 0.00	0.43 / 0.1
**Nausea**	3/3.2	10.6/15.3	89.56 / 84.70	7.35 / 12.20	2.02 /2.50	0.53 / 0.40	0.53 / 0.30
**Cough**	1.9/1.6	11.1/6.1	88.60 / 98.90	9.27 / 0.80	1.60 / 0.30	0.11 / 0.00	0.43 / 0.00
**Stomach pain**	3.8/0.5	11.6/5.7	88.82 / 94.30	7.24 / 5.20	2.34 / 0.30	1.06 / 0.10	0.53 / 0.10
**Dizziness**	3.7/3.2	13.1/11.0	86.58 / 89.00	10.12 / 7.80	2.02 / 1.90	0.85 / 0.80	0.43 / 0.50
**Flu-like**	4/7.3	14/18.9	85.30 / 81.10	10.22 / 11.60	2.88 / 3.60	1.17 / 1.90	0.43 / 1.90
**Chills**	5.8/4.9	15.9/10.3	84.24 / 89.70	10.01 / 5.40	3.73 / 2.10	1.28 / 2.00	0.75 / 0.80
**Sleep problems**	6.5/3.4	19.2/15.5	80.40 / 84.50	12.89 / 12.00	4.47 / 2.20	1.49 / 1.20	0.75 / 0.00
**Joint pains**	5.7/7.7	19.2/20.2	79.66 / 79.80	14.16 / 12.60	4.15 / 4.10	1.49 / 2.10	0.53 / 1.50
**Headache**	8.5/8.1	24.9/20.9	73.16 / 79.10	17.25 / 12.80	6.39 / 4.60	2.24 / 2.10	0.96 / 1.30
**Weakness**	10.2/13.8	32.8/37.3	66.35 / 62.70	22.79 / 23.50	7.45 / 7.70	2.56 / 3.40	0.85 / 2.60
**Muscular pain**	12.8/14.9	36.2/37.3	64.22 / 62.70	22.26 / 22.40	9.27 / 8.30	2.77 / 4.60	1.49 / 2.00
**Feeling tired**	17.9/22.2	45/45.2	54.74 / 54.8	26.73 / 23.0	12.57 / 12.80	4.37 / 6.5	1.60 / 2.9
**Red/swollen arm injection site**	22.9/28.8	59.5/63.8	41.53 / 36.20	36.74 / 34.90	14.27 / 19.40	5.43 / 6.70	2.02 / 2.60

Additional Analyses

The additional analyses focused on three issues. The first goal was to further analyze sub-groups of side-effects to discern if the current findings were specific to certain side-effect clusters. The primary division of side-effects was based on earlier research which showed that psychological factors predicted a narrow list of seven side-effects [3], out of which, three side-effects were observed in the Pfizer trials [4]. Accordingly, the data were re-analyzed to examine if vaccine hesitancy predicting side-effects would hold across different side-effects (See Tables S3 and S4). The second goal was to assess if the effect of vaccine hesitancy predicting side-effects would hold beyond different vaccine hesitancy items, i.e., even vaccine hesitancy items that do not tap expectations of side-effects. Showing that results hold even beyond these expectations items would suggest that results are not stemming from domain overlap, i.e., that the vaccine hesitancy items tapping side-effects are driving the prediction of actual side-effects, please see Table S5. We also addressed in a separate analysis only the (three) vaccine hesitancy expectation items (see Table S6). The third goal was to address if this effect would hold when general anxiety disorder symptoms (GAD-7) and a single W2 item measuring vaccine expectation, were added (see Table S7).

1.Side-effect division

The 7 side-effects used in a previous study [3] were 1) pain at injection site, 2) fever, 3) chills, 4) headache, 5) joint pain, 6) nausea, and 7) fatigue. Three of these side-effects (pain at injection site, headaches and fatigue) were selected due to their importance in clinical trials [4] and appear on the CDC site [5]. We added weakness as another side-effect related to the first group of side-effects, yielding 8 side-effects (this did not change results). The results appear in Table S3, which show that the W1-to W2 cross hesitancy effect was even slightly stronger for the non-typical side-effects (.20 vs. .15, See Table S3).

Table S3, Model results for different groupings of side-effects (a group of seven side-effects vs. remaining side-effects).

	χ^2(df)	p	CFI	TLI	RMSEA
**Model based on the following side-effects: pain at injection site, fever, chills, headache, joint pain, nausea, fatigue (based on [3])**	.156 (1)	.693	1.000	1.022	.02 (.000,.071)
Side-effects (lagged effect) .35***					
Hesitancy to Side-effects .15***					
Hesitancy (lagged effect) .53***					
Side-effects to Hesitancy -.04 p=.211					
**Multi-Gender group analysis (male/female)**	4.73(2)	.094	.995	.929	.041 (.00, .09)
Side-effects (lagged effect) .28 ***/ .37***					
Hesitancy to Side-effects. 28*** /.09*					
Hesitancy (lagged effect) .53***/.54***					
Side-effects to Hesitancy .01 p=.897 / .04 p=.270					
**Model based on the remaining side-effects: swollen lymph nodes, flu like symptoms, Face paralysis, allergy, dizzy, itchy throat, swollen eyes, intestine problems, vomiting, cough, rash, and sleeping problems**	.160 (1)	.693	1.000	.1031	.02 (.000,.071)
Side-effects (lagged effect) .14***					
Hesitancy to Side-effects .20***					
Hesitancy (lagged effect) .55**					
Side-effects to Hesitancy -.04 p=.221					
**Multi-Gender group analysis (male/female)**	4.73(2)	.094	.994	.903	.040 (.01, .08)
Side-effects (lagged effect) .08 p = .131 / .24***					
Hesitancy to Side-effects. 29** /.12*					
Hesitancy (lagged effect) .54***/.55***					
Side-effects to Hesitancy -.05 p=.328 / -.03 p=.423					

*$p<.01$, **$p<.001$***$p<.0001$, *p* value depicted when not significant.

Table S4 shows that result held even when side-effects only included the three side-effects obtained in clinical Pfizer Trials [see 4], namely, headaches, fatigue and pain at injection site were. Similar to Table S3, effects seem even stronger in the larger group of side-effects (.20 vs. .15).

Table S4, Model results for different groupings of side-effects (a group of three side-effects vs. remaining side-effects).

	χ^2(df)	p	CFI	TLI	RMSEA
**Model based on three side-effects: headaches, fatigue and pain at injection site (Based on [4])**	.156 (1)	.693	1.000	1.022	.02 (.000,.071)
Side-effects (lagged effect) .37***					
Hesitancy to Side-effects .15***					
Hesitancy (lagged effect) .54***					
Side-effects to Hesitancy .03 p=.386					
**Multi-Gender group analysis (male/female)**	4.73(2)	.094	.995	.926	.043 (.00, .088)
Side-effects (lagged effect) .31 ***/ .37***					
Hesitancy to Side-effects .22*** /.11*					
Hesitancy (lagged effect) .54***/.54***					
Side-effects to Hesitancy -.02 p=.745 / .03 p=.398					
**Model based on the remaining side-effects: fever, chills, joint pain, nausea, swollen lymph nodes, flu like symptoms, Face paralysis, allergy, dizzy, itchy throat, swollen eyes, intestine problems, vomiting, cough, rash, and sleeping problems**	.160 (1)	.693	1.000	.1031	.02 (.000,.071)
Side-effects (lagged effect) .14***					
Hesitancy to Side-effects .20***					
Hesitancy (lagged effect) .55**					
Side-effects to Hesitancy -.04 p=.221					
**Multi-Gender group analysis (male/female)**	4.73(2)	.094	.995	.918	.043 (.000, .094)
Side-effects (lagged effect) .16** / .31***					
Hesitancy to Side-effects. 34*** /.10*					
Hesitancy (lagged effect) .54***/.55***					
Side-effects to Hesitancy -.02 p=.643 /.01 p=.786					

*p<.01, **p<.001***p<.0001, p value depicted when not significant.

2. Additional analyses addressing vaccine hesitancy items

These supplementary findings are similar to the results depicted in the text, i.e., this was true across the different groupings of side-effects. Namely, only hesitancy predicted subsequent side-effects but not the reverse. Similar results were obtained when the model was run on each of the five factors (obtained after running a factor analysis on side-effects). The aim of the next analyses was to examine if results also remain the same when removing vaccine hesitancy items related to side-effects. This analysis was performed twice, once after removal of items that even indirectly pertained to expectations that the vaccine is harmful (items 1,2,3,5 & 8, Table S5, top), and again a second time, after removing only three items directly pertaining to negative expectations of vaccination side-effects (Table S5, bottom). As shown in Table S5, the expected results were obtained.

Table S5. Model results when vaccine items pertaining to side-effects were removed.

	χ^2(df)	p	CFI	TLI	RMSEA
**Model based on vaccine hesitancy items - that did not even indirectly address expectations about being harmed by the vaccine (without items, 1,2, 3, 5, and 8)**	.156 (1)	.693	1.000	1.044	.02 (.000,.071)
Side-effects (lagged effect) .30***					
Hesitancy to Side-effects .09**					
Hesitancy (lagged effect) .44***					
Side-effects to Hesitancy -.03 p=.417					
**Multi-Gender group analysis (male/female)**	4.73 (2)	.094	.991	.866	.043 (.000, .094)
Side-effects (lagged effect) .23*** / .36***					
Hesitancy to Side-effects. 19*** /.07 p = .126					
Hesitancy (lagged effect) .42***/.46***					
Side-effects to Hesitancy -.04 p=.463 /-.02 p=.582					
**Model based on vaccine hesitancy without the three items (without items 1,2, & 8) that directly tap side-effects**	.156 (1)	.693	1.000	.1032	.00 (.000,.071)
Side-effects (lagged effect) .31***					
Hesitancy to Side-effects .12***					
Hesitancy (lagged effect) .50***					
Side-effects to Hesitancy -.02 p=.578					
**Multi-Gender group analysis (male/female)**	4.73 (2)	.094	.993	.901	.043 (.000, .094)
Side-effects (lagged effect) .21*** / .35***					
Hesitancy to Side-effects. 23*** /.08 p = .091					
Hesitancy (lagged effect) .48***/.52***					
Side-effects to Hesitancy -.04 p=.424 /-.01 p=.863					

p*<.01, ***p*<.001**p*<.0001, *p* value depicted when not significant.

As indicated, our goal (Table S5) was to examine if vaccine hesitancy prospectively predicts side-effects. We also wanted to show that it was not the component of negative expectation (regarding side-effects) which was driving the link between vaccine hesitancy and actual side-effects. Had this above analysis not been conducted, it could have been claimed that the linking of vaccine hesitancy (which includes expectations of side-effects) with actual side-effects, stems from the domain overlap, as both variables are tapping aspects of side-effects. In summary, the results of the main model reported in the text were reliable across side-effects and across vaccine hesitancy items, namely, for all cases, vaccine-hesitancy predicted subsequent side-effects across different vaccine hesitancy items and different side-effects; the opposite direction was not obtained in any scenario.

3. Expectation and Anxiety

One final issue is that Nocebo effects may typically be impacted by one's expectations [6] and one's negative affect [7]. As mentioned in the limitations (see Discussion), we did not measure expectations of specific outcomes, as typically computed. Thus, in the next analysis we addressed only the three vaccine hesitancy items that tap general negative expectations to asses if results would remain the same. These data appear in Table S6, and show the same result pattern. Only the direction of vaccine hesitancy predicting side-effects was significant, but not the opposite direction.

Table S6. Model results for vaccine hesitancy items that solely pertain to expectations.

**Model based only on the three vaccine hesitancy items (1,2,8) - that tap negative expectations/side-effects**	.156 (1)	.693	1.000	1.024	.000 (.000, .071)
Side-effects (lagged effect) .26***					
Hesitancy to Side-effects .22***					
Hesitancy (lagged effect) .51***					
Side-effects to Hesitancy 05 p=.093					
**Multi-Gender group analysis (male/female)**	4.73 (2)	.094	.995	.926	.043 (.000, .018)
Side-effects (lagged effect) .18** / .33***					
Hesitancy to Side-effects. 38*** /.12*					
Hesitancy (lagged effect) .52***/.48***					
Side-effects to Hesitancy .03 p=.538 /.07 p=.107					

*p<.01, **p<.001***p<.0001, p value depicted when not significant.

We employed a final model that includes both general anxiety disorder symptoms (GAD-7) assessed at both waves, to examine for example if it predicts side-effects. We also measured a single item at W2 that assess one's general expectation "that the vaccine will

protect me from COVID-19". This item was rated on a 5-point Likert scale 1-not at all to 5-very much). We entered this item in the final analysis as a dependent variable to be predicted by W1 variables (e.g., if expectation is predicted by anxiety).

Table S7. Model results after including general anxiety symptoms (GAD-7 scores from both W1 and W2) and a single W2 expectation item.

	χ^2(df)	p	CFI	TLI	RMSEA
General full sample model	14.79 (9)	.097	.994	.975	.029 (.000,.055)
Side-effects (lagged effect) .27***					
Hesitancy to Side-effects .18***					
Hesitancy (lagged effect) .54***					
Side-effects to Hesitancy .01 p=.813					
GAD-7 (lagged effect) .34***					
Hesitancy to GAD-7. 14***					
Side-effects to GAD-7 .10**					
GAD-7 to Hesitancy -.01 p=.834					
GAD-7 to side-effects .02 p=.567					
Side-effects to Expectation .02 p=.624					
Hesitancy to Expectation -.28***					
Multi-Gender group analysis (male/female)	25.78 (18)	.105	1.000	1.022	.024 (.000,.043)
Side-effects (lagged effect) .18 ***/ .34***					
Hesitancy to Side-effects. 34*** /.10*					
Hesitancy (lagged effect) .53***/.54***					
Side-effects to Hesitancy -.02 p=.631 / .02 p=.554					
GAD-7 (lagged effect) .25 ***/ .41***					
Hesitancy to GAD-7. 19*** /.11*					
Side-effects to GAD-7 .10 p=.088 / .09*					
GAD-7 to Hesitancy .01 p=.880 / -.02 p=.656					
GAD-7 to side-effects -.01 p=.866 / .02 p=.693					
Side-effects to Expectation .04 p=.514 / .00 p=.964					
Hesitancy to Expectation -.27*** / -.28***					

p*<.01, ***p*<.001**p*<.0001, *p* value depicted when not significant.

The result pattern was the same even when adding both this single expectation item and the GAD-7 score, as integral components of the cross-lagged model. Namely, vaccine hesitancy predicted side-effects but not the opposite. Additionally, vaccine hesitancy and side-effects at W1 predicted more GAD-7 symptoms at W2, above and beyond the lagged association of the GAD-7 symptoms over time. However, GAD-7 symptoms did not predict subsequent hesitancy or side-effects. Regarding the W2 expectation item, side-

effects at W1 did not predict one's expectation (that the vaccine will protect me from COVID-19), however more hesitancy at W1 predicted lower levels of this expectation. The results were also similar in the multigroup model.

In conclusion, the supplementary results indicate that the data reported in the main text were reliable both across different groupings of side-effects and different groupings of vaccine hesitancy items (expectancy items vs. non-expectancy items). These results held even when anxiety was entered into the model. In all cases, vaccine hesitancy predicted future side-effects, beyond other effects, such as one's previous experience reflected by W1 side-effects. The opposite direction of W1 side-effects predicting W2 vaccine hesitancy was not significant in any scenario.

References

1. Giambi C, Fabiani M, D'Ancona F, et al. Parental vaccine hesitancy in Italy: Results from a national survey. Vaccine 2018;36:779–787.
2. Palgi Y, Bergman YS, Ben-David B, et al. No psychological vaccination: Vaccine hesitancy is associated with negative psychiatric outcomes among Israelis who received COVID-19 vaccination. J Affect Disord 2021;287:352-3.
3. Geers AL, Clemens KS, Faasse K, et al. Psychosocial Factors Predict COVID-19 Vaccine Side-effects. Psychother and Psychosom 2021;4:1-3.
4. Polack FP, Thomas SJ, Kitchin N, Absalon J, Gurtman A, Lockhart S, et al.. Safety and efficacy of the BNT162b2 mRNA COVID-19 vaccine. N Engl J Med. 2020 Dec;383(27):2603–15.
5. https://www.cdc.gov/coronavirus/2019-ncov/vaccines/expect/after.html
6. Lindheimer, J. B., Szabo, A., Raglin, J. S., Beedie, C., Carmichael, K. E., & O'Connor, P. J. (2020). Reconceptualizing the measurement of expectations to better understand placebo and nocebo effects in psychological responses to exercise. *European journal of sport science*, *20*(3), 338-346.
7. Geers, A. L., Faasse, K., Guevarra, D. A., Clemens, K. S., Helfer, S. G., & Colagiuri, B. (2021). Affect and emotions in placebo and nocebo effects: What do we know so far?. *Social and Personality Psychology Compass*, *15*(1), e12575
